# Supplementary material for: A Label-Free Proteomic Approach for the Identification of Biomarkers in the Exosome of Endometrial Cancer Serum
Source: Cancers (Basel). 2022 Dec 19;14(24):6262. doi: 10.3390/cancers14246262 (PMC9776976; doi:10.3390/cancers14246262)

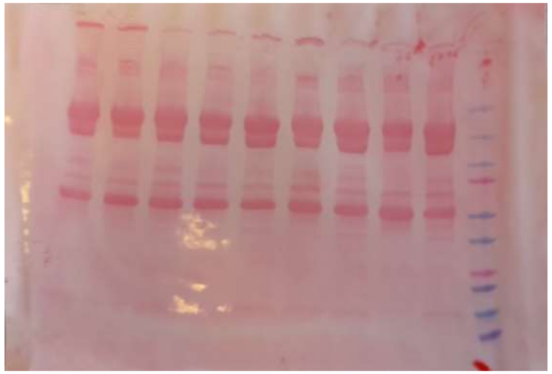

Gel 4

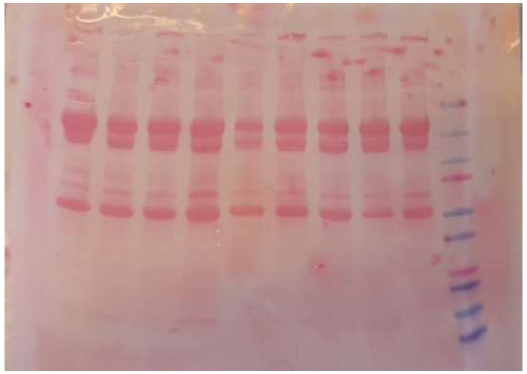

Gel 3

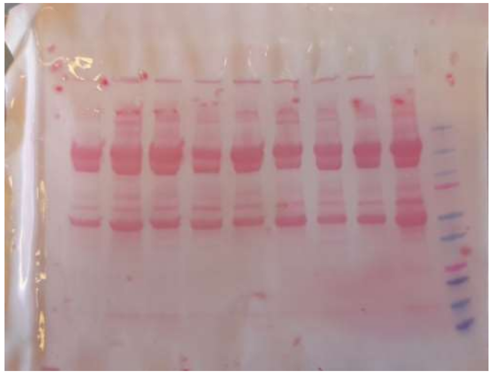

Gel 2

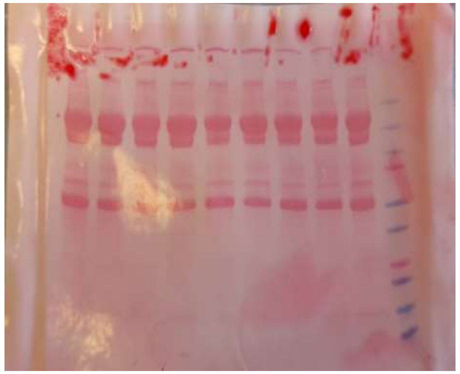

Gel 1

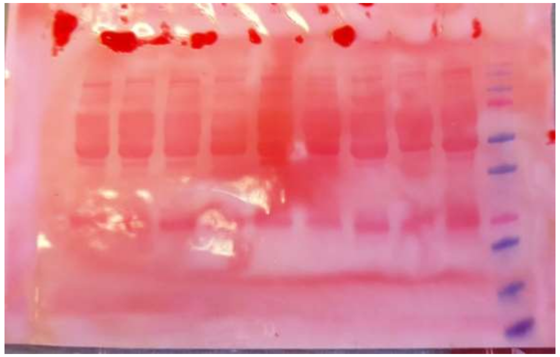

Gel 8

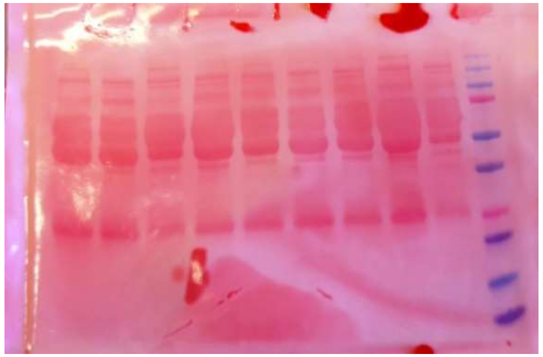

Gel 7

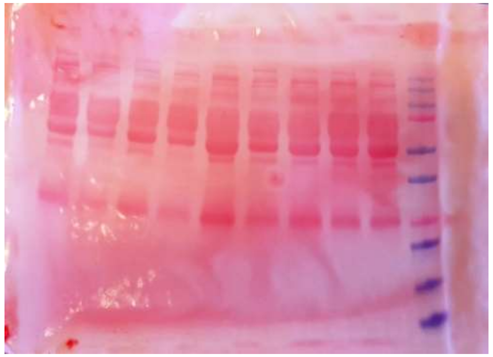

Gel 6

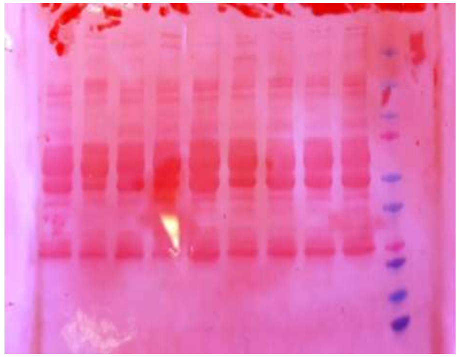

Gel 5

Whole membrane stained with red ponceau

Whole membrane of CD9 and CD63 proteins

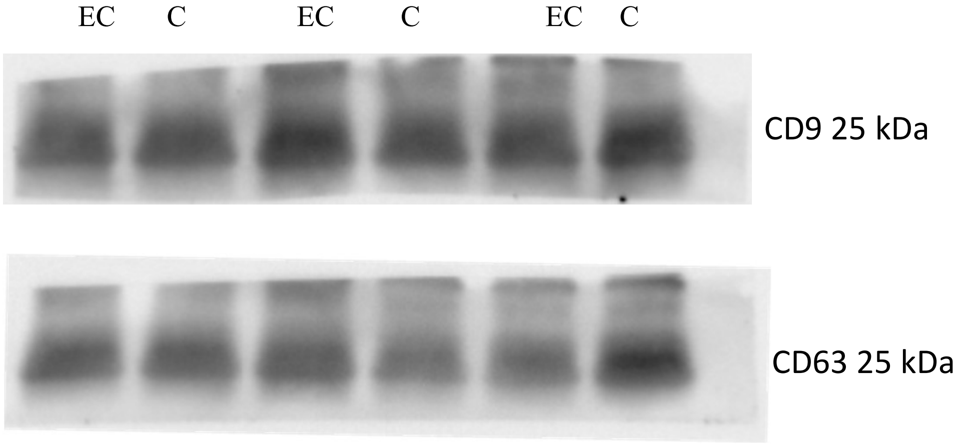

C-control
EC- endometrial cancer

Whole membrane of APOA1 protein

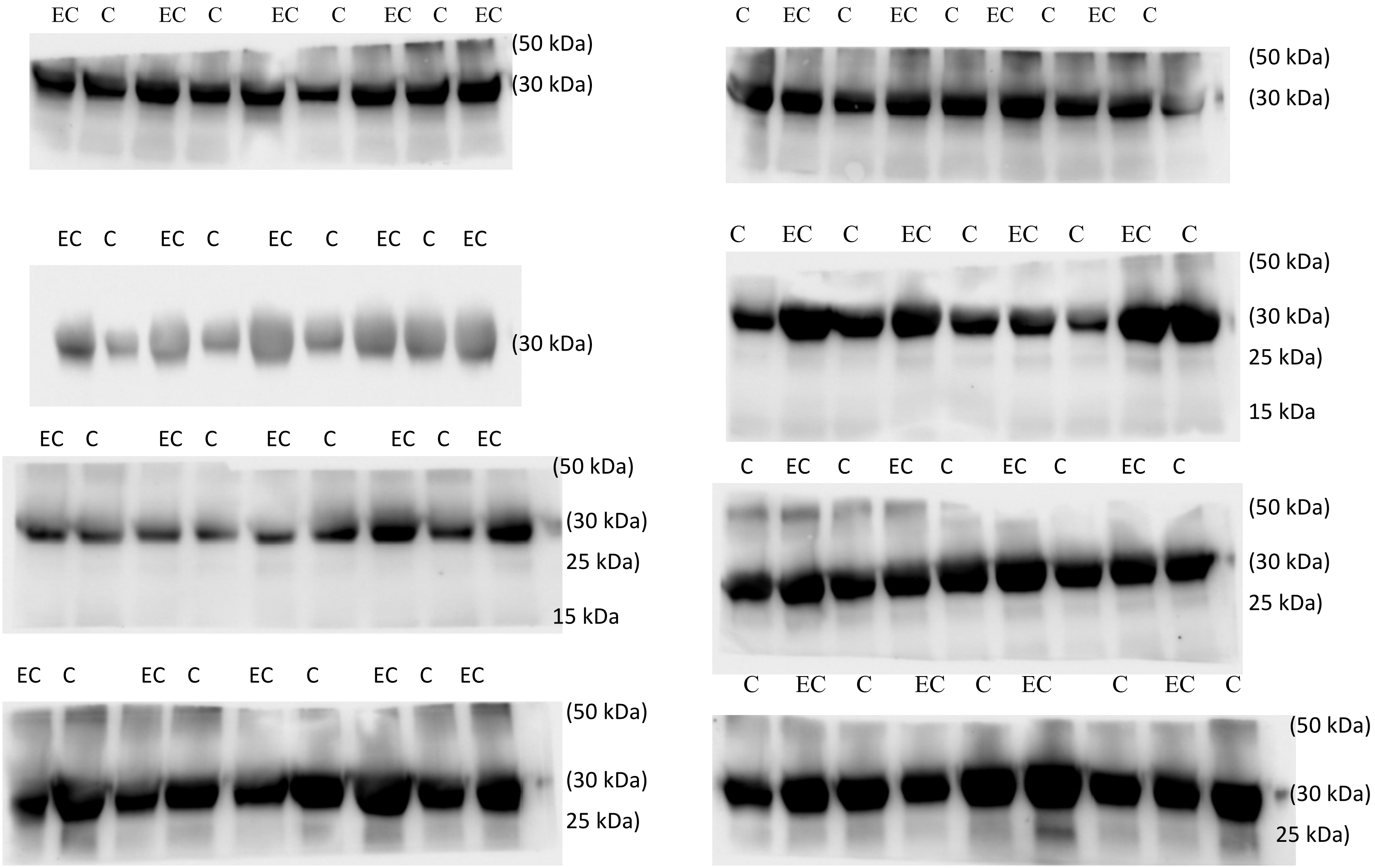

Whole membrane of HBB protein

C-control
EC- endometrial cancer

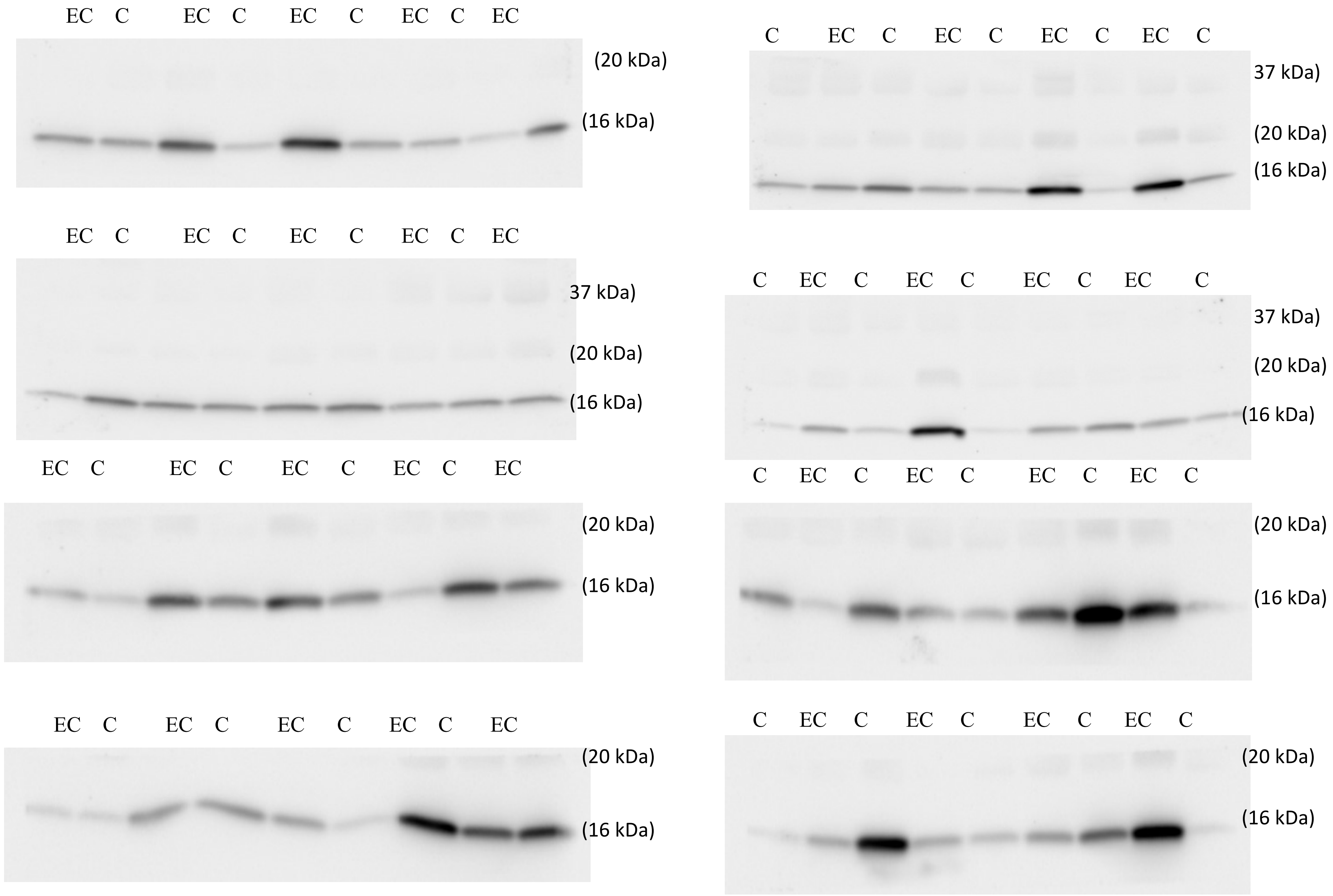

Whole membrane of CA1 protein

C-control
EC- endometrial cancer

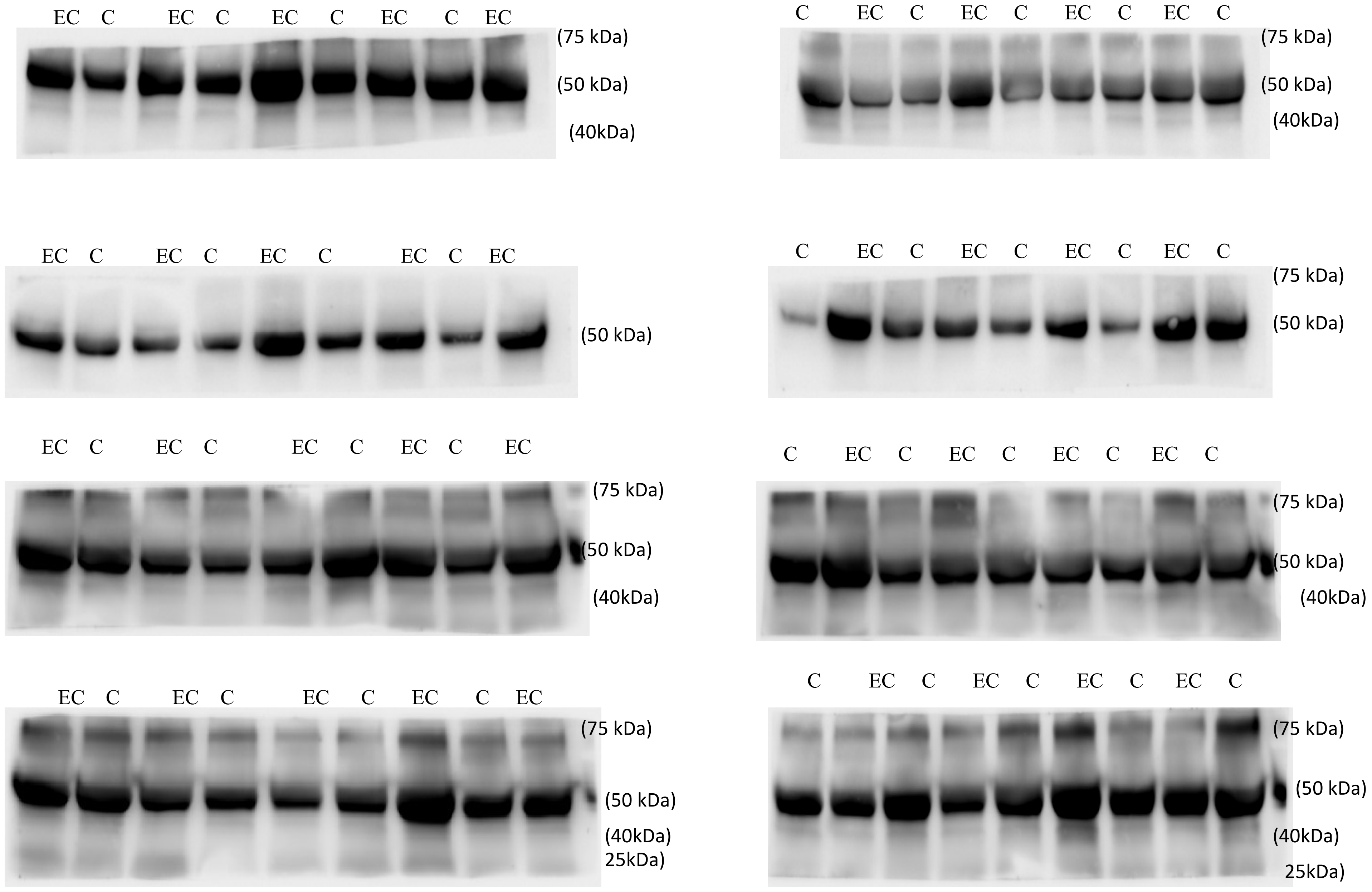

Whole membrane of HBD protein

C-control
EC- endometrial cancer

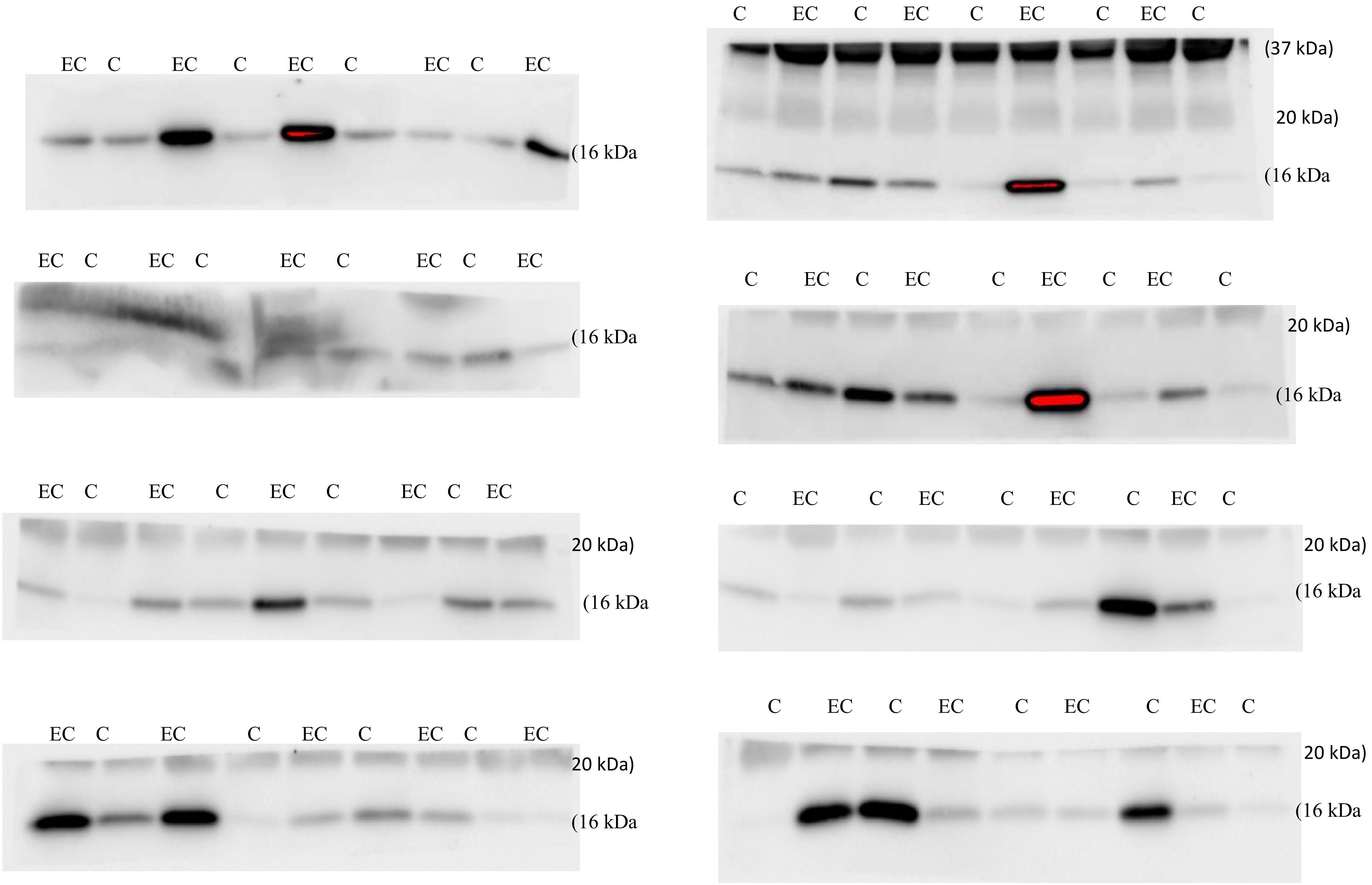

C-control
EC- endometrial cancer

Whole membrane of LPA protein

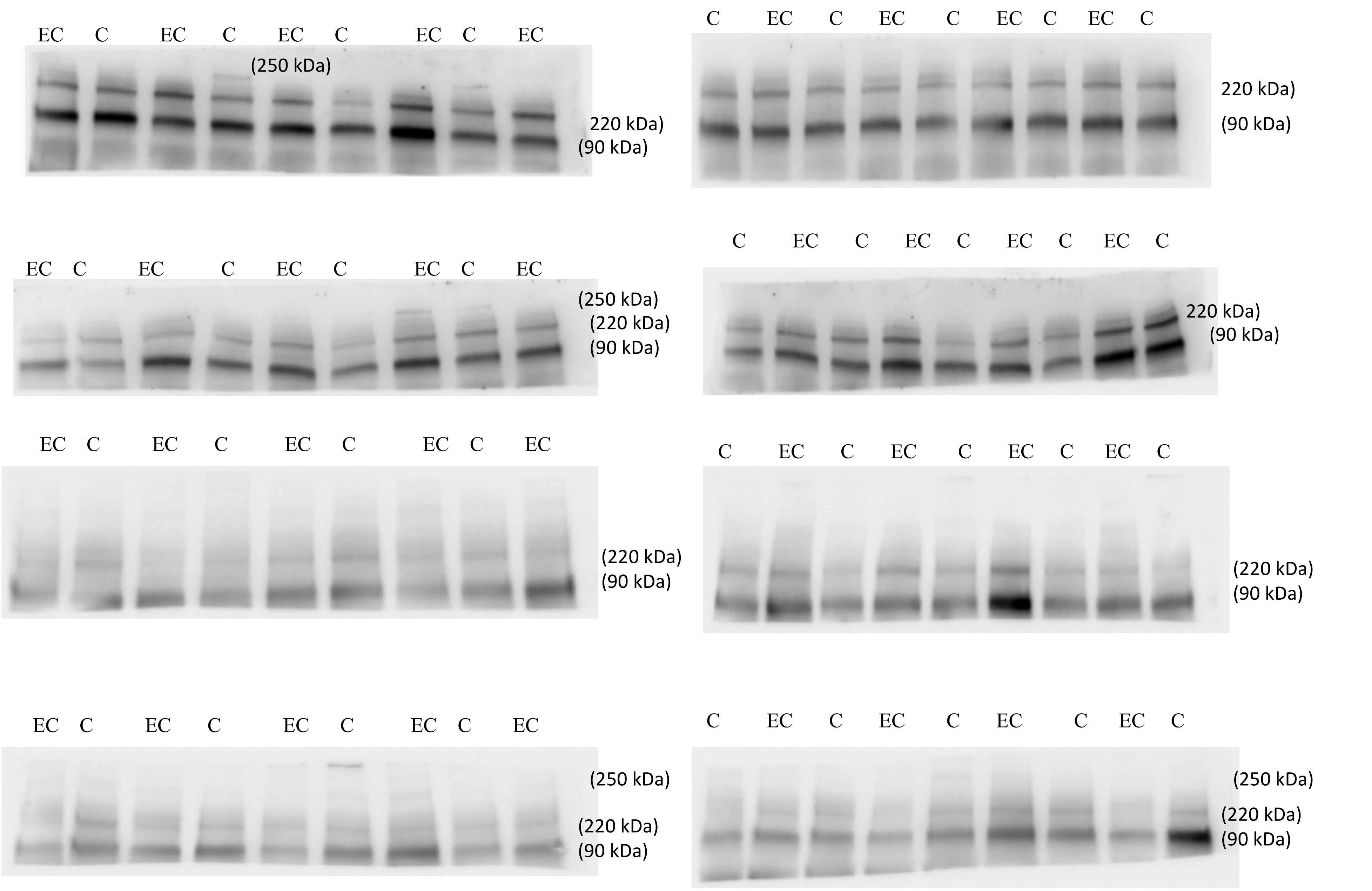

Whole membrane of SAA4 protein

C-control
EC- endometrial cancer

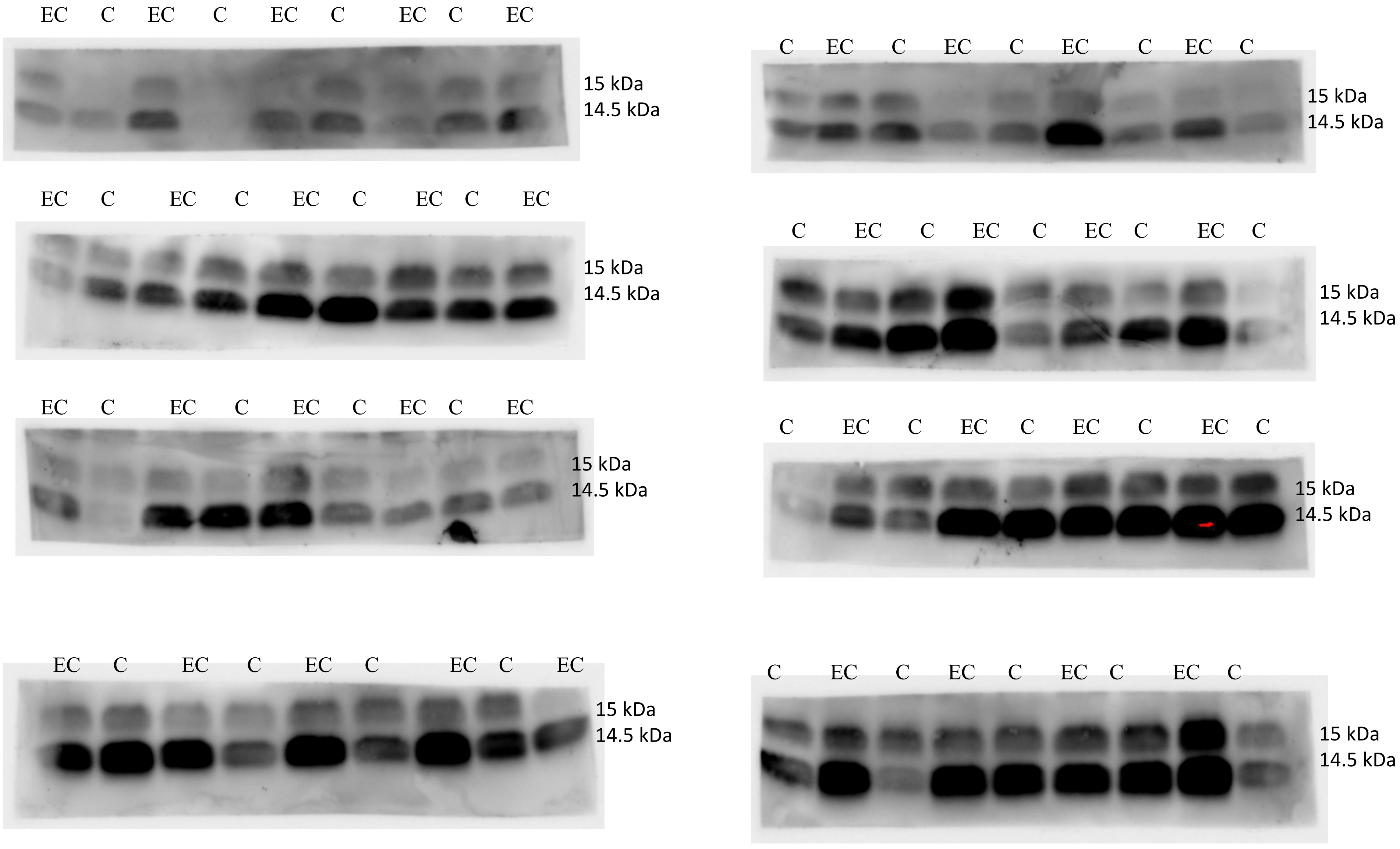

Whole membrane of PF4V1 protein

C-control
EC- endometrial cancer

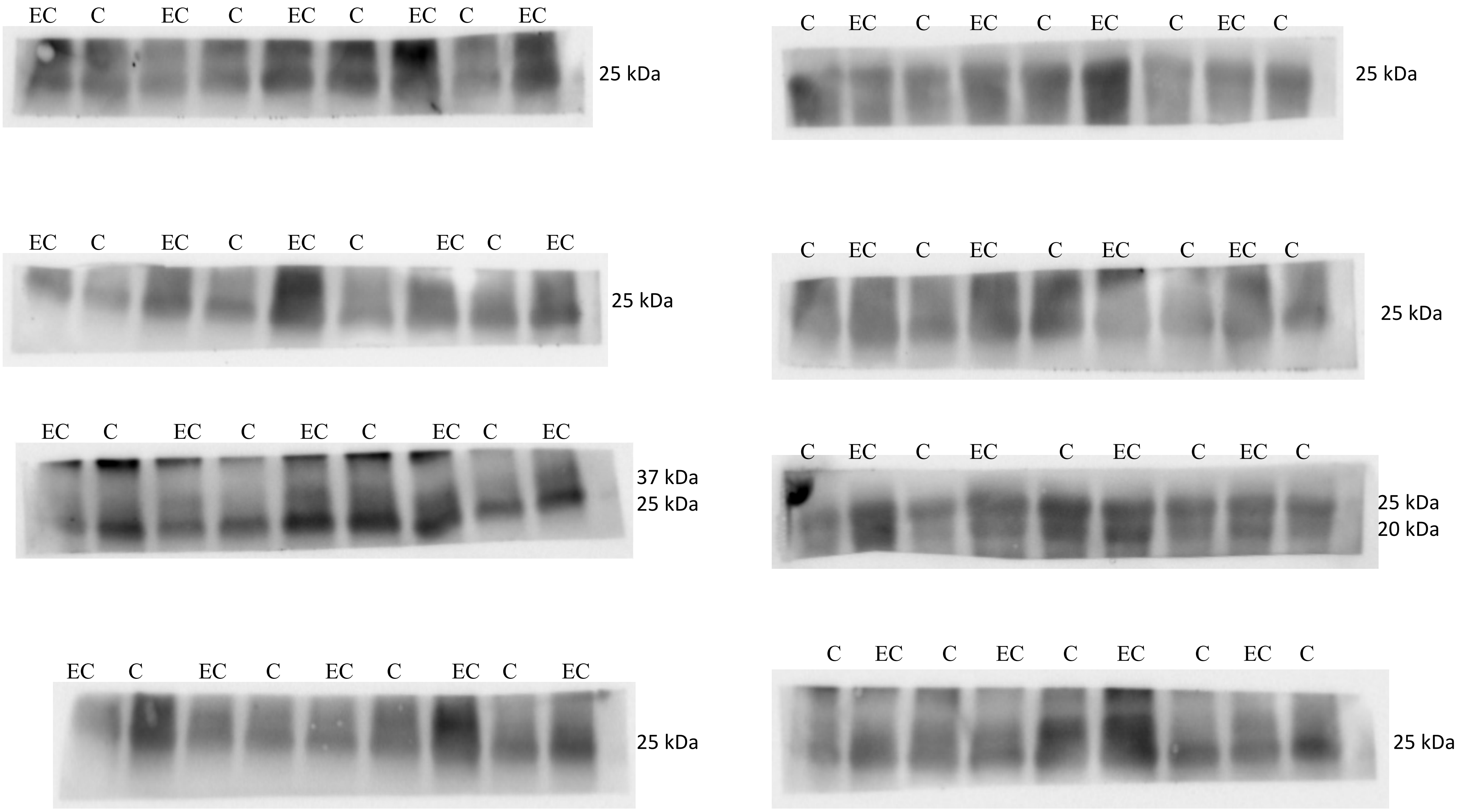

Whole membrane of APOE protein

C-control
EC- endometrial cancer

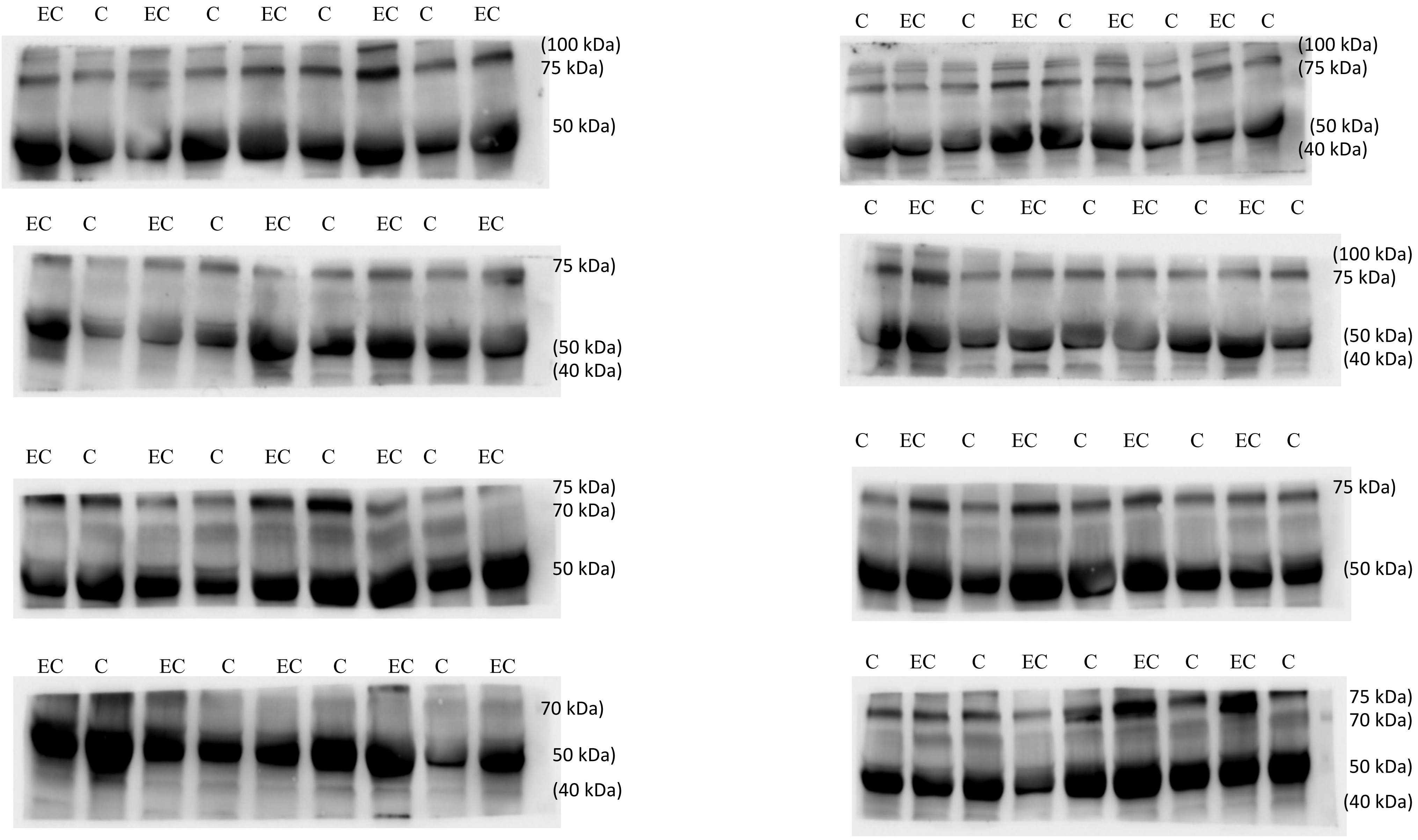

Supplement: Supplementary file 1 [file cancers-14-06262-s001.zip › cancers-1971544- supplementary/Figure s1.pdf]
